# Bioactive Hydrogel Dressings Incorporating Decellularized Ovine Liver Extracellular Matrix (ECM) for Effective Wound Regeneration

**DOI:** 10.1155/bmri/4345216

**Published:** 2026-09-12

**Authors:** Hadis Doosti, Parisa Mohammadi, Sara Darakhshan, Mohsen Zhaleh, Hadi Hossainpour, Faranak Aghaz, Reza Tahvilian

**Affiliations:** ^1^ Pharmaceutical Sciences Research Center, Health Institute, Kermanshah University of Medical Sciences, Kermanshah, Iran, kums.ac.ir; ^2^ Medical Biology Research Center, Health Technology Institute, Kermanshah University of Medical Sciences, Kermanshah, Iran, kums.ac.ir; ^3^ Department of Molecular Medicine, School of Medicine, Kermanshah University of Medical Sciences, Kermanshah, Iran, kums.ac.ir; ^4^ Department of Biology, Faculty of Science, Razi University, Kermanshah, Iran, razi.ac.ir; ^5^ Department of Anatomical Sciences, School of Medicine, Kermanshah University of Medical Sciences, Kermanshah, Iran, kums.ac.ir; ^6^ Behbahan Faculty of Medical Sciences, Behbahan, Iran, modares.ac.ir; ^7^ Nano Drug Delivery Research Center, Health Technology Institute, Kermanshah University of Medical Sciences, Kermanshah, Iran, kums.ac.ir

**Keywords:** bioactive material, carboxymethyl cellulose (CMC), decellularization, polyethylene glycol (PEG), sodium alginate (SA), tissue regeneration, wound dressing

## Abstract

Chronic wounds present significant healthcare challenges, necessitating advanced biomaterials for effective tissue regeneration. This study introduces a bioactive hydrogel dressing incorporating decellularized ovine liver‐derived extracellular matrix (LdECM), a biologically active material containing structural proteins, glycosaminoglycans, and matrix‐associated regenerative cues that may support cellular infiltration and tissue regeneration. Tannic and gallic acids were added for antimicrobial and antioxidant properties while enhancing mechanical strength and hydrogel stability. Characterization studies verified that the hydrogel possessed mechanically adaptive stability along with an appropriate porous architecture. Mechanical evaluation demonstrated that Construct C exhibited a Young′s modulus of 0.2382 MPa, indicating suitable mechanical stability while maintaining the flexibility required for wound‐dressing applications. In vitro analyses further showed that Construct C had superior cytocompatibility, achieving 127% cell viability after 24 h, as well as notable antibacterial activity against *Staphylococcus aureus* and *Escherichia coli*, with inhibition zones measuring 1.6 and 1.3 mm, respectively. In vivo investigations using a rat wound model revealed enhanced wound healing performance, with Constructs C and D attaining approximately 70% wound closure by Day 14 and demonstrating superior healing performance compared with the other experimental groups. Moreover, LdECM‐containing constructs consistently outperformed LdECM‐free formulations, highlighting the synergistic contribution of extracellular matrix incorporation and polymer cross‐linking. Despite rapid initial degradation, the hydrogels retained bioactivity, supporting tissue remodeling. Whereas the negative control group showed comparable wound closure, histological analysis revealed inferior skin quality. In contrast, LdECM‐based constructs resulted in fully developed skin layers with proper collagen organization and vascularization. These findings support the use of LdECM as a biologically active component in wound‐dressing design.

## 1. Introduction

Wound healing is a multifactorial biological process that is of great importance for restoring skin integrity and function after injury [1]. This crucial process is divided into four stages: homeostasis, inflammation, proliferation, and remodeling, each of which depends on the highly integrated actions of many cell populations and signaling mechanisms [2–4]. Whereas many wounds progress naturally through the stages of normal wound healing, this natural process does not occur in chronic wounds caused by diabetes, venous insufficiency, or infection. Chronic wounds pose significant clinical challenges, including prolonged patient suffering, increased risk of infection, and increased healthcare costs [1]. Prolonged wound healing and poor wound healing conditions remain a major challenge in contemporary treatment methods.

Despite the benefits of traditional hydrogels in providing a moist environment, their clinical efficacy is often limited by a lack of intrinsic bioactive cues necessary for chronic wound healing. These materials promote efficient healing by mimicking the natural extracellular matrix (ECM) and providing moisture that are utilized by cell migration and tissue repair [5]. Their inherent biocompatibility reduces the risk of adverse reactions during the healing process. In addition, hydrogels can be filled with bioactive agents, growth factors, and even pharmaceuticals. This allows for customizable properties to enhance therapeutic outcomes. The adaptability to wound surfaces makes them effective in preventing infection while allowing oxygenation and nutrition [6].

Bioactive components play a critical role in improving wound healing by enhancing cellular and molecular activities necessary for tissue repair. These components mimic the natural ECM and create a bioactive environment that actively supports regeneration. Specifically, bioactive materials stimulate processes such as chemotaxis, neocollagenesis, and angiogenesis, the formation of new vascular networks essential for nutrient and oxygen delivery [7]. Incorporation of bioactive signals, such as growth factors, cytokines, and antioxidant agents, into biomaterials may further activate endothelial cells, modulate immune responses, and develop vascular networks responsible for the wound healing process. In addition, these agents act as regulators of immune cell activity, attracting neutrophils and macrophages to the wound site, while releasing growth factors that shift the healing process to the proliferative phase, which is ensured by keratinocyte migration, fibroblast activation, and granulation tissue formation [3].

Decellularized ECM is a biomaterial that retains the structural and biochemical properties of the ECM after removal of cellular components. Acting as a scaffold containing collagen, glycosaminoglycans (GAGs), and growth factors integral to cellular communication and tissue regeneration, decellularized ECM closely mimics the native tissue microenvironment. This scaffold encourages cellular adhesion, proliferation, and differentiation [8, 9]. The biocompatibility and structural integrity of decellularized ECM derived from various tissues, including the urinary bladder, skin, and cardiac tissue, have led to their extensive investigation as scaffolds for wound healing applications. Recent advances in automated decellularization technologies have further improved process reproducibility and scaffold quality by enabling objective monitoring of ECM preservation and decellularization efficiency [10]. Nevertheless, owing to the distinct advantages of liver‐derived extracellular matrix (LdECM), it may offer superior performance in regenerative medicine. The LdECM supports robust vascularization due to its enrichment in angiogenic factors such as vascular endothelial growth factor (VEGF) and hepatocyte growth factor (HGF) [11–13]. Indeed, chronic skin wounds are often ischemic, making the liver′s high angiogenic factor content a logical solution to revascularize the wound bed [14]. The rationale for selecting LdECM is supported by previous studies demonstrating its regenerative potential beyond hepatic applications. Bobrova et al. reported that scaffolds incorporating decellularized liver‐derived microparticles enhanced fibroblast adhesion and proliferation, promoted cutaneous wound healing, and supported functional skin regeneration, including hair follicle formation [15]. Similarly, Panahi et al. demonstrated that decellularized liver matrices preserve a bioactive microenvironment capable of supporting cellular adhesion, proliferation, and tissue regeneration [16]. These findings support the rationale for incorporating LdECM into the present wound dressing as a biologically active component rather than functioning solely as a passive structural scaffold. In addition, compared with the denser matrices obtained from other organs, its highly porous and intrinsically vascularized architecture can facilitate enhanced cellular infiltration and improved oxygen diffusion. Moreover, the preservation of cytokines and chemokines that regulate and orchestrate immune responses represents a notable immunomodulatory advantage, alongside the direct interaction of ECM proteins with immune cell receptors, which collectively contribute to the attenuation of excessive inflammation and foreign body responses [17]. When combined with the abundance and cost‐effectiveness of ovine liver as a xenogeneic source, these attributes suggest that LdECM may represent a promising and relatively underexplored biomaterial for wound healing applications. However, its utility in bioactive wound dressings remains insufficiently investigated and warrants further evaluation. In this study, we developed a multifunctional bioactive platform by integrating LdECM into a composite matrix of carboxymethyl cellulose (CMC), sodium alginate (SA), and polyethylene glycol (PEG). This innovative approach combines the structural stability and hydration capacity of CMC and SA with the proangiogenic and bioactive characteristics of LdECM. Recent studies have highlighted the potential of biomimetic scaffolds to enhance the retention and regenerative effects of bioactive secretomes, supporting the synergistic role of scaffold‐based delivery systems in wound healing [18]. We hypothesized that the bioactive and proregenerative characteristics of LdECM, combined with the antioxidant and antimicrobial properties of tannic acid (TA) and gallic acid, would synergistically accelerate functional wound healing rather than simple contraction. To test this, the constructs were comprehensively characterized for their physicochemical properties, followed by in vitro fibroblast assays and in vivo evaluation in a rat excision model to distinguish between simple wound closure and high‐quality tissue regeneration.

## 2. Materials and Methods

### 2.1. Materials

Formaldehyde solution, sodium hydroxide (NaOH), hydrochloric acid (HCl), xylazine, ketamine hydrochloride (ketamine HCl), pepsin powder (porcine gastric mucosa), hematoxylin and eosin (H&E) staining kit, Masson′s trichrome (MT) staining kit, 3‐(4,5‐dimethylthiazol‐2‐yl)‐2,5‐diphenyltetrazolium bromide (MTT reagent), phosphate‐buffered saline (PBS), and crystal violet stain were obtained from Sigma‐Aldrich Company (United States). Phenol red powder, fetal bovine serum (FBS), and Dulbecco′s Modified Eagle Medium/Nutrient Mixture F‐12 (DMEM/F‐12) medium were obtained from Gibco (Thermo Fisher Scientific Inc.). Sodium dodecyl sulfate (SDS), Triton X‐100, acetic acid, CMC, PEG‐400, TA, gallic acid, and calcium chloride (CaCl_2_) were purchased from Merck (Germany). SA was obtained from CARLO ERBA (France).

### 2.2. Preparation of Dressing Constructs

#### 2.2.1. Decellularization of Ovine Liver

Fresh ovine liver was acquired from a local supplier and subjected to a decellularization process designed to eliminate cellular components while preserving the ECM, as previously published [19], with slight alterations. Given that liver tissue is highly vascularized, it is essential to wash the tissue prior to the decellularization procedure. Consequently, the liver was meticulously sliced into thin layers and immersed in sterile distilled water (DW) for 15 min, a process that was repeated three times. Subsequently, the small pieces of liver were immersed in a 1% SDS solution for a duration of 48 h. During the SDS rinses, the solution was changed after 24 h. Following this treatment, the tissue was rinsed with DW and then subjected to immersion in a 1% Triton X‐100 solution for a period ranging from 1 to 3 h, after which it was again rinsed with DW. To ensure the thorough removal of all detergents, the decellularized tissues were stirred in DW for an additional 24 h. Characterization of the decellularized tissue was conducted using histological staining methods, specifically H&E to validate the removal of cell nuclei and MT staining for remaining collagen content. Stained samples were examined under a light microscope (Olympus, BX51).

#### 2.2.2. Preparation of LdECM Gel

Following characterization, the decellularized ovine liver (LdECM) was subjected to freeze‐drying to achieve a stable powder. For this purpose, the tissue was initially frozen at −80°C and subsequently lyophilized under reduced pressure for a duration of 24 h. The resulting freeze‐dried LdECM was stored at −20°C until further use. To prepare the gel at a final concentration of 5 mg/mL, the powdered LdECM was dissolved in an appropriate amount of 0.5 N acetic acid; then, 1% (w/v) pepsin powder was added, and the mixture was stirred at 4°C for 72 h. After digestion, the resultant gel was sterilized with an ultraviolet (UV) chamber (UV Crosslinker, QIAGEN) and was stored at 4°C until further use. Prior to use, the gel was neutralized through the gradual addition of 5 N NaOH, adjusting the pH to physiological levels. Neutralization was monitored by the color change of phenol red (to which some of its powder was added) from yellow to pink.

#### 2.2.3. Synthesis of Bioactive Wound Dressings

The preparation of the dressing constructs involved the formulation of a composite matrix using equal solutions of 4% (w/v) Alg and 2% (w/v) CMC, 20 mL each, along with 1 mL of PEG‐400 to achieve a homogeneous mixture. For constructs that incorporated TA and gallic acid, 0.4 and 0.2 g of each compound, respectively, were added to the mixture and stirred to ensure complete solubilization. This formulation was then transferred to a petri dish and incubated at 37°C for 24 h to facilitate the drying process.

Following the initial drying period, a solution of 1% CaCl_2_ was applied as a cross‐linking agent to the dried constructs (5 mL for each petri dish). The petri dishes were then allowed to rest at room temperature for 30 min to enable effective cross‐linking. Subsequently, the constructs were washed once with DW to remove any unreacted CaCl_2_. Then, 20 mL of the neutralized gel was introduced into each of the prepared compositions. The dishes were then returned to the incubator for an additional 24 h to ensure ideal integration of the gel with the underlying matrix. Upon completion of the drying phase, the petri dishes containing the dressings were transferred into propylene bags and stored at 4°C to maintain their structural integrity and bioactive properties until further analysis.

Four distinct types of bioactive wound dressings were created. The first dressing, called Construct A, was made entirely of cross‐linked polymers. Constructs B, C, and D included the prepared LdECM gel within the polymer matrix, with Constructs C and D specifically containing TA and gallic acid (Table 1).

**Table 1:** Composition and characteristics of the prepared bioactive hydrogel constructs (A–D).

Construct	Composition	Presence of LdECM	Cross‐linkers/agents	Added bioactive compounds
A	CMC + SA + PEG	✗	Ca^2+^	None
B	A + LdECM	✓	Ca^2+^	None
C	A + LdECM	✓	Ca^2+^	Tannic acid
D	A + LdECM	✓	Ca^2+^	Gallic acid

*Note:* Construct A served as a polymer‐only control, whereas Constructs B–D incorporated liver‐derived ECM (LdECM) within the polymeric matrix. Constructs C and D were further modified with tannic acid and gallic acid, respectively.

Abbreviations: CMC, carboxymethyl cellulose; LdECM, liver‐derived extracellular matrix; PEG, polyethylene glycol; SA, sodium alginate.

### 2.3. Characterization of Dressing Constructs

#### 2.3.1. Swelling Ratio

The solvent absorption capacity of the constructs was assessed through a swelling ratio test. We cut our constructs into 1 × 1 cm pieces, dried them, and recorded their dry weights (*W*_*d*_). The dried samples were then placed in test tubes with 10 mL of PBS at 37°C. After soaking for intervals of 30 min, 1 h, 2 h, and 24 h, the samples were taken out, blotted with filter paper to eliminate surface moisture, and weighed again to find their swollen weight (*W*_*s*_). The swelling rate (SR) was calculated using Equation (1):

(1) SR=Ws−WdWd.

#### 2.3.2. Degradation Rate

The degradation rate test was conducted using samples that matched the size of those used in the swelling ratio test. Samples (from Constructs B–D) were immersed in PBS for an in vitro biodegradability assessment, and their initial weights (*W*_0_) were recorded. Each sample was placed in 10 mL of PBS solution in a test tube and incubated at 37°C while shaking at 80 rpm for 1, 2, 3, and 4 days, respectively. After these time intervals, the specimens were taken out of the PBS, dried in an oven at 60°C, and their weights were measured (*W*_*t*_). The remaining mass percentage relative to the initial dry weight was then calculated using Equation (2):

(2) Remaining weight%=WtW0×100.

#### 2.3.3. Fourier‐Transform Infrared (FTIR) Spectroscopy

FTIR spectroscopy was utilized with an FTIR spectrometer (Shimadzu Corporation, Model IRPrestige‐21) to examine the functional groups and cross‐linking interactions in polymeric constructs. The analysis was conducted in two stages: prior to and following cross‐linking (for constructs before the addition of ECM), allowing for a comprehensive understanding of structural changes. FTIR spectra were obtained with samples prepared in KBr pellet format to ensure precision. The data were analyzed to pinpoint absorption bands associated with CMC, PEG, and alginate, along with any shifts that signified successful cross‐linking.

#### 2.3.4. Scanning Electron Microscopy (SEM)

The microstructural characteristics of the bioactive wound dressings were examined using an SEM set to an accelerating voltage of 5.0 kV. To prepare the samples (Constructs B, C, and D, both with and without LdECM) for imaging, the following steps were taken: The constructs were immersed in liquid nitrogen to preserve their structural integrity and were then sectioned to expose the cross‐sectional area. The sectioned samples were affixed to the SEM stage using a conductive adhesive to ensure stability during imaging. To minimize charging artifacts and improve image quality, the surfaces of the dehydrated samples were coated with a thin layer of gold using a sputter coater. The SEM images were acquired at magnifications of 3000x and 5000x. The resulting micrographs were analyzed to identify changes in microstructural features, such as porosity, fiber alignment, and surface morphology, which could affect the performance of the wound dressing.

#### 2.3.5. Mechanical Properties

The mechanical properties of the constructs were assessed using a universal testing machine (UTM). The scaffolds were prepared as standardized specimens with dimensions of 20 × 10 × 0.5 mm and subjected to uniaxial tension at a constant strain rate of 2 mm/min. Stress–strain curves were recorded, and important mechanical parameters such as ultimate tensile strength, modulus of elasticity, and elongation at break were determined. Each type of scaffold was tested three times to ensure consistent results.

#### 2.3.6. Cytocompatibility

The cytocompatibility of the synthesized polymeric constructs was assessed through two primary assays: the MTT assay and the scratch assay with crystal violet staining, focusing on fibroblast cell viability and proliferation, respectively [17, 20]. The human skin fibroblast cell line HSF‐PI 16 (female, derived from normal skin tissue; RRID:CVCL_9V77; NCBI Code: C192) was obtained from the National Cell Bank of Iran, Pasteur Institute of Iran (Tehran, Iran), through a collaborator, prior to the experiments described herein. Authentication of the cell line was performed by the National Cell Bank of Iran prior to distribution using short tandem repeat (STR) profiling (multiplex PCR analysis of 13 CODIS core STR loci plus amelogenin for gender confirmation), showing a complete match with the reference profile (no discrepancies reported). This cell line is not listed as misidentified or contaminated in the ICLAC register of misidentified cell lines. The cells were confirmed to be free of mycoplasma contamination by routine testing at the National Cell Bank of Iran (using PCR‐based methods, including real‐time PCR as reported by the bank) prior to distribution, and no mycoplasma contamination was detected in the cultures used for the described experiments. The fibroblast cell line (HSF‐PI 16) was cultured in DMEM/F‐12 medium supplemented with antibiotics and 10% FBS under controlled conditions (5% CO_2_ at 37°C). The cells were maintained until reaching 70%–80% confluence, and the culture medium was refreshed every 2 days. Subsequently, the cells were trypsinized and seeded into 96‐well plates at a density of 5 × 10^3^ cells per well.

MTT assay: To assess cell viability, cells at approximately 70% confluence were treated with media exposed to Constructs A–D (0.03 g/mL) for 24 h. Untreated cells were considered the negative control. Then, cells were washed with sterile PBS and incubated with 50 *μ*L of MTT dye (5 mg/mL in PBS) for 4 h at 37°C in a 5% CO_2_ atmosphere in the dark. Following the removal of excess unreacted dye, DMSO (200 *μ*L/well) was added to dissolve the formazan crystals. The absorbance was measured at 570 nm using a spectrophotometer, and cell viability was calculated based on the absorbance ratio of treated to control groups.

Scratch assay: To evaluate the effects of the constructs on cell proliferation, the scratch assay was conducted. Fibroblast cells were seeded into 6‐well plates and allowed to grow until they reached confluence. A straight‐line scratch was created in each well using a yellow sampler tip, establishing a “wound” area. The cells were then treated with media exposed to Constructs A–D for 24 h, whereas a control group without treatment was also included. Following, cells were washed with PBS and stained with crystal violet solution for 20 min at 37°C. The stained cells were then observed using an inverted optical microscope (OPTIKA SN263975, Italy), and the cells were photographed to assess cell migration and proliferation into the scratched area. This allowed for qualitative analysis of how treatment with the polymeric constructs influenced fibroblast proliferation and migration rates.

Each assay included appropriate controls and replicates to ensure statistical validity.

#### 2.3.7. Antibacterial Efficacy

The antibacterial activity of the constructs against *Staphylococcus aureus* ATCC 25923 (Gram‐positive) and *Escherichia coli* ATCC 25922 (Gram‐negative) was determined using the disk diffusion method with Mueller–Hinton agar (MHA) medium. Agar plates were prepared by dissolving 38 g of the medium in 1 L of DW and then autoclaved. Subsequently, the solution was poured onto the plates and allowed to solidify. Thereafter, 100 *μ*L of the prepared bacterial suspension (10^8^ CFU/mL) was spread onto an agar plate. The constructs (B, C, and D) were then cut into circles with a diameter of 2 cm, placed on the agar plate, and incubated at 37°C for 24 h. The inhibition zone around each sample, indicating the absence of bacterial growth, was measured using a vernier caliper and recorded in millimeters. Gentamicin (20 *μ*g/disk) disks were tested as positive controls using the disk diffusion method.

### 2.4. In Vivo Wound Healing Studies

#### 2.4.1. Animal Modeling and Ethical Considerations

A total of 32 adult male Wistar rats, each weighing between 200 and 220 g (aged 6 weeks), were acquired from Kermanshah University of Medical Sciences (KUMS). The rats were kept under standardized conditions, with food and water provided ad libitum. They were housed in individual cages at a controlled room temperature of 22°C ± 0.5°C, subjected to a 12‐h light/12‐h dark cycle. All experimental procedures received approval from the Ethics Committee at KUMS (IR.KUMS.AEC.1403.012). The rats were randomly allocated into six distinct groups to assess wound healing dynamics, as follows: Group 1, wound receiving no treatment (negative control group, *n* = 5); Group 2, wound treated with Hartmann Comfeel (positive control group, *n* = 5); Group 3, wound treated with Construct A (*n* = 6); Group 4, wound treated with Construct B (*n* = 6); Group 5, wound treated with Construct C (*n* = 6); and Group 6, wound treated with Construct D (*n* = 6).

To establish a standardized wound model, a circular incision (1 cm in diameter) was carefully excised from the dorsal surface of each rat. Prior to the surgical procedure, the animals were anesthetized using a combination of ketamine HCl (50 mg/kg) and xylazine (5 mg/kg), injected intraperitoneally, to ensure humane treatment and minimize distress. Following anesthesia, the fur in the designated wound region was shaved to facilitate optimal visibility and access, after which the wound was induced, and immediate treatment was initiated.

#### 2.4.2. Wound Closure Measurements

To provide a visual record of the healing process, photographic documentation of the wounds was captured on Days 1, 4, 7, 10, and 14. The dressings applied to the wounds were changed every 3 days, specifically on Days 4, 7, and 10, and were secured in place using adhesive tape to maintain consistency and sterility. The diameter of the wounds was measured using ImageJ software, and the rate of wound healing was assessed using Equation (3):

(3) Wound contraction %=D0−DtD0×100,

where *D*_0_ represent the diameter of the wound on the day of wound induction and *D*_*t*_ represent the wound diameter on day *t*.

#### 2.4.3. Histological Analysis

At the completion of the 14‐day observation period, a histological dissection was conducted, wherein samples of the healed skin from the wound area were fixed in formalin fixative. In parallel, samples of the uninjured skin were collected at the time of wounding and also subjected to formalin fixation. The euthanasia of the rats was carried out using a suitable anesthetic protocol that ultimately led to the stopping of vital functions.

Paraffin blocks were prepared from the fixed tissue specimens, which were subsequently sectioned to facilitate slide preparation. The histological analysis incorporated two staining techniques: H&E and MT, with the resultant slides being examined under a light microscope to elucidate the histopathological characteristics associated with the wound healing process. Various parameters were evaluated by an expert histologist to assess wound healing, including epithelial regeneration, granulation tissue thickness, the count of fibroblasts, macrophages, and neutrophils, as well as the assessment of angiogenesis at the wound site.

### 2.5. Statistical Analysis

All data were analyzed using GraphPad Prism V.8.0 software (GraphPad Software Inc., San Diego, California). Comparative evaluations among groups were analyzed using one‐way ANOVA, and a *p* value of ≤ 0.05 was statistically significant.

## 3. Results

### 3.1. Decellularization and Characterization of LdECM

**Figure 1:**
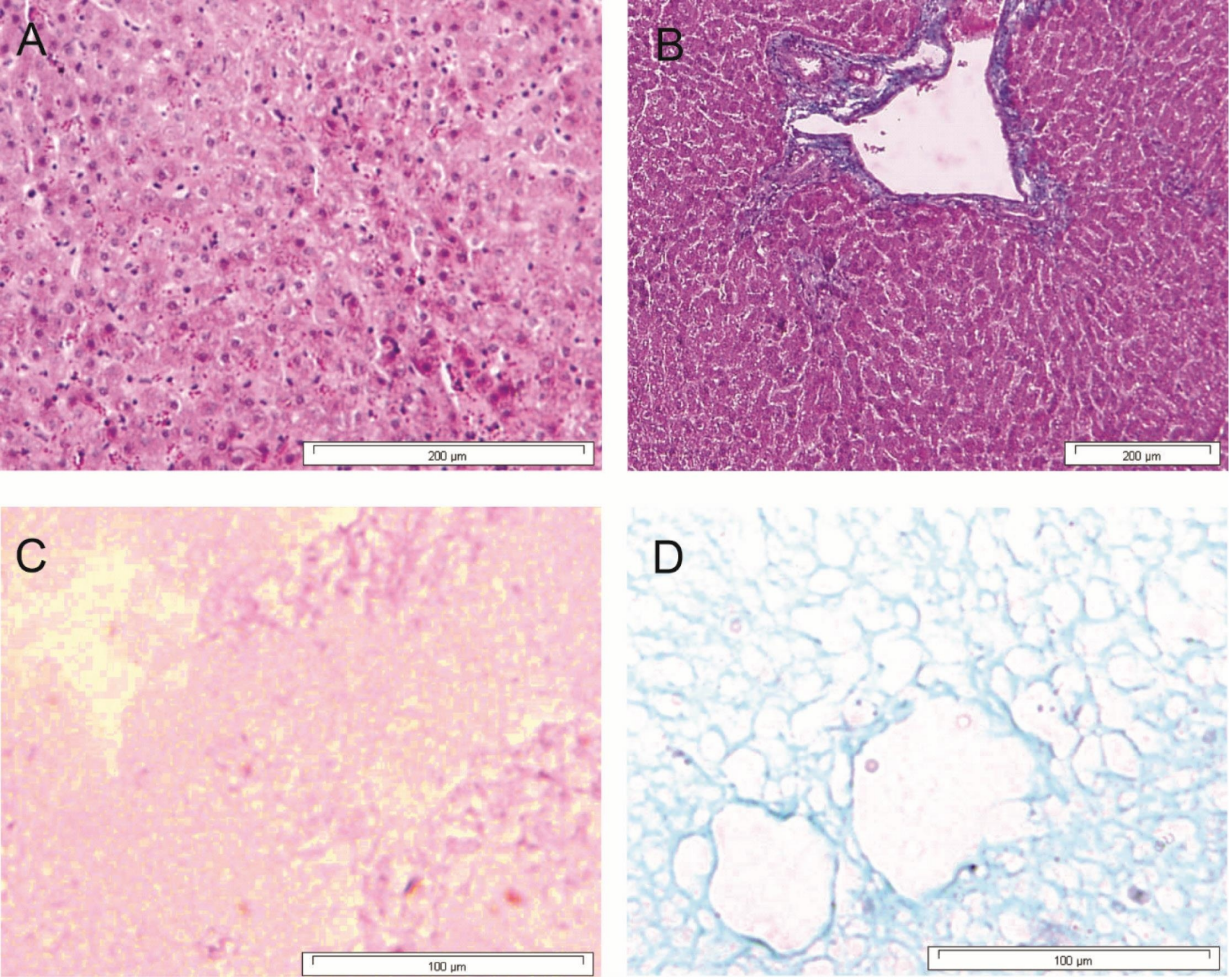
Histological evaluation of native and decellularized ovine liver scaffolds. (A) Hematoxylin and eosin (H&E) staining of native ovine liver tissue. (B) Masson′s trichrome (MT) staining of native tissue, highlighting collagen deposition (blue). (C) H&E staining of decellularized liver scaffold, demonstrating complete cell removal. (D) MT staining of decellularized scaffold, showing preserved collagen architecture.

The ovine liver was decellularized using a chemical‐based protocol, resulting in a visibly more transparent appearance compared with the native tissue. Histological analysis of H&E‐stained sections confirmed the successful removal of cellular components, as evidenced by the absence of cell nuclei in the decellularized liver, unlike the native liver tissue (Figure 1A,B). Furthermore, MT staining demonstrated the preservation of the LdECM, with blue‐stained regions indicating the presence of collagen fibers (Figure 1C,D). Given that the accuracy and precision of this decellularization technique have been extensively validated in previous studies [19, 21], only histological staining was employed in the current study to confirm effective decellularization.

#### 3.1.1. Swelling Behavior

**Figure 2:**
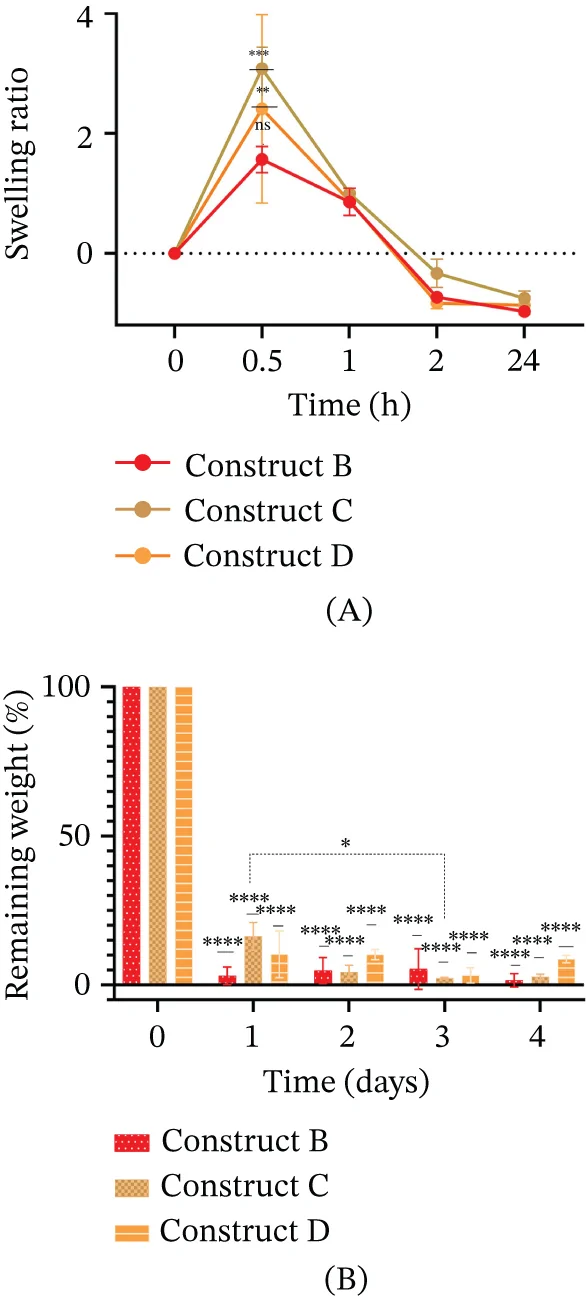
Swelling and degradation profiles of hydrogel constructs. (A) Swelling kinetics of Constructs B, C, and D over time. (B) Weight loss of constructs in PBS over 4 days. Data presented as mean ± SD (*n* = 3). Significance:  ^∗^ *p* < 0.05,  ^∗∗^ *p* < 0.01,  ^∗∗∗^ *p* < 0.001, and  ^∗∗∗∗^ *p* < 0.0001; ns = nonsignificant.

This study investigated the swelling behavior of the constructs in PBS to evaluate their water absorption capacity under conditions that simulate wound exudate. All constructs reached their maximum swelling within 30 min. Among the tested groups, Constructs C and D exhibited significantly greater swelling compared with Construct B. Following this peak (0.5 h), a marked reduction in swelling was observed across all groups by 2 h. At this time point, the swelling ratio approached baseline levels and remained relatively stable up to 24 h. Furthermore, statistical analyses of the intergroup differences are presented in Figure 2A.

#### 3.1.2. Degradation Behavior

The degradation behavior of the constructs was assessed to evaluate weight loss over time, focusing on Constructs B, C, and D. As shown in Figure 2B, all constructs exhibited substantial degradation, with the highest weight loss occurring on the first day. Constructs B, D, and C demonstrated respective weight loss values of 96.89%, 89.7%, and 83.7%. Degradation progressed steadily until the fourth day, with minor fluctuations that were not statistically significant. Detailed statistical analyses and related information are provided in Figure 2B.

#### 3.1.3. FTIR Analysis

**Figure 3:**
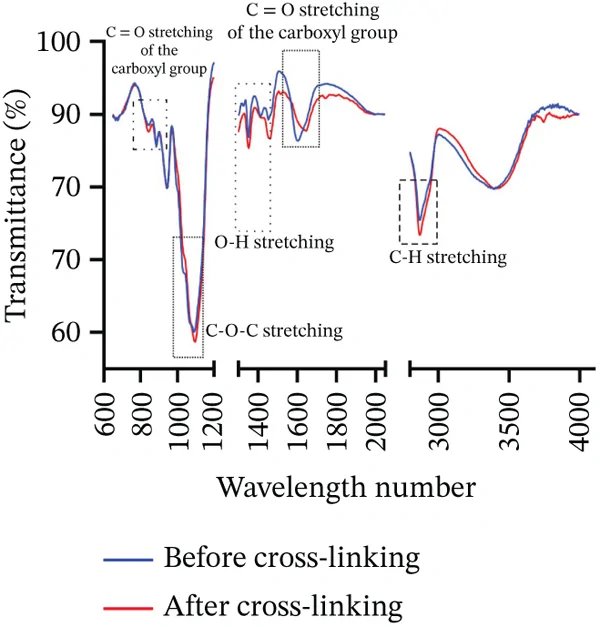
FTIR spectral analysis of hydrogels before and after cross‐linking (without LdECM). The blue spectrum represents the uncross‐linked hydrogel, showing characteristic peaks at 3390 cm^−1^ (O–H stretching), 2864 cm^−1^ (C–H stretching), 1597 cm^−1^ (C=O stretching), and 1085 cm^−1^ (C–O–C stretching). The red spectrum represents the postcross‐linking hydrogel, demonstrating shifts to 1641 cm^−1^ (C=O) and 1099 cm^−1^ (C–O–C), along with the appearance of a new peak at 1737 cm^−1^, indicative of covalent bonding and ionic cross‐linking.

The FTIR spectral analysis of the uncross‐linked and Ca^2+^‐cross‐linked blended hydrogels is shown in Figure 3. Key peaks were observed in the fingerprint regions specific to the hydrogel constituent compounds. The broad and overlapping absorption peaks indicated effective mixing and interaction among the polymeric components (SA, CMC, and PEG). A broad and intense absorption band is observed in the 3200–3600 cm^−1^ region, centered at approximately 3400 cm^−1^, corresponding to the O–H stretching vibrations of the hydroxyl groups present in the polymer chains [22]. After cross‐linking, the asymmetric –COO^-^ stretch shifted to a higher wavenumber (1650 cm^−1^), accompanied by a blue shift in the C–H stretching region. A redshift was also seen for the PEG etheric link (from 1105 to 1098). Furthermore, a peak shift from 1069 to 1061 cm^−1^ was observed, indicating changes in the skeletal environment of the pyranose rings in CMC and SA. The C–H stretching peak at ~2920 cm^−1^ and the C–O–C ether bands (~1100 cm^−1^) remain largely unchanged in position, though minor intensity variations are observed. No new covalent peaks (e.g., ester bands at ~1740 cm^−1^) were detected.

#### 3.1.4. SEM Analysis

**Figure 4:**
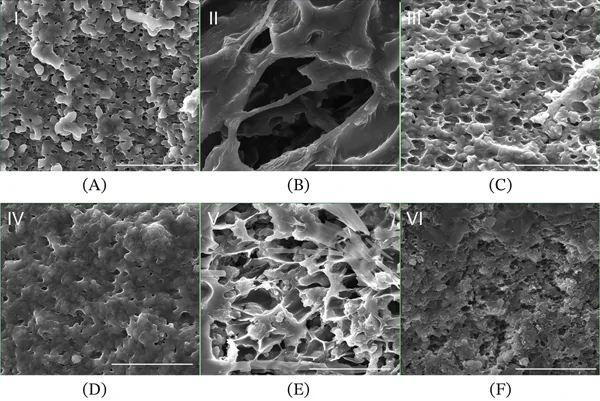
SEM microstructural analysis of hydrogel constructs with and without LdECM. (A–C) Drug‐free constructs: (A) Construct B (SA/CMC/PEG) has moderate porosity with irregular pores. (B) Construct C (tannic acid) exhibits more uniform pores and organization. (C) Construct D (gallic acid) displays a smoother, compact morphology. (D–F) LdECM‐containing counterparts: (D) Construct B with LdECM reveals significantly increased pore size and interconnectivity via ECM fibers. (E) Construct C with LdECM maintains enhanced porosity with some tannic acid–induced compaction. (F) Construct D with LdECM shows substantially increased porosity and heterogeneity, overcoming gallic acid′s compactness. LdECM incorporation universally enhances porosity and structural complexity, potentially facilitating cellular infiltration.

The cross‐sectional SEM analysis of constructs, revealing microstructural differences arising from variations in their compositions, is presented in Figure 4A–F. The drug‐free construct (Construct B without LdECM) exhibited moderate porosity characterized by irregular and relatively loose pores, which is typical of SA/CMC/PEG‐based hydrogels. Upon incorporation of LdECM (Construct B with LdECM), pore size and interconnectivity increased markedly, with ECM fibers forming a more open and interconnected internal network that more closely mimics native biological architecture. Construct containing TA (Construct C without LdECM) displayed more uniform pore morphology and a comparatively organized structure, indicative of enhanced cross‐linking and moderate matrix densification. Its LdECM‐containing counterpart (Construct C with LdECM) preserved the increased porosity imparted by LdECM incorporation, although TA still induced a discernible degree of compaction within the fibrous network. Construct formulated with gallic acid (Construct D without LdECM) exhibited a smoother and mildly compact morphology with fewer interconnected pores, consistent with the weaker cross‐linking capacity of gallic acid. Following LdECM incorporation (Construct D with LdECM), both porosity and structural heterogeneity increased substantially, whereas a mild degree of surface uniformity associated with gallic acid was retained. Overall, LdECM incorporation enhanced porosity and internal structural complexity across all formulations, a feature critical for facilitating improved cellular infiltration. Furthermore, LdECM contributed to modulating the extent of matrix compaction induced by each polyphenolic cross‐linker.

#### 3.1.5. Mechanical Properties

**Figure 5:**
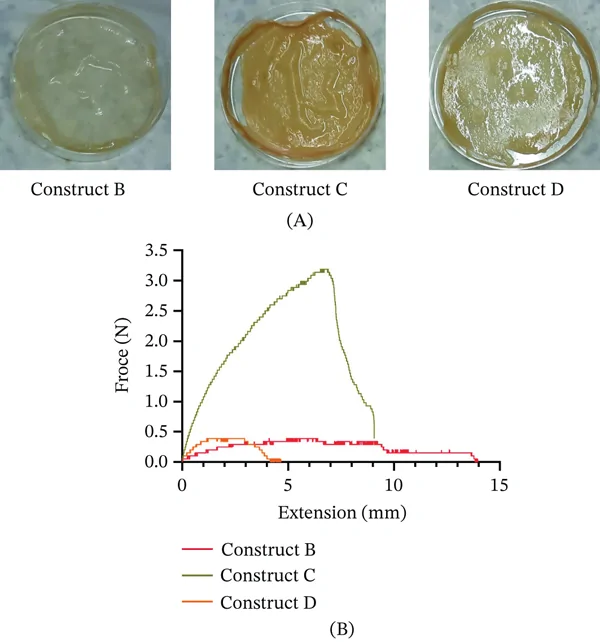
Macroscopic appearance and tensile behavior of hydrogel constructs. (A) Representative images of Constructs B, C, and D show distinct visual differences related to polyphenol incorporation, with Constructs C and D exhibiting darker coloration and more compact surfaces. (B) Tensile force–extension curves highlight clear mechanical contrasts: Construct C demonstrates markedly higher tensile strength and extension capacity, indicating enhanced cross‐linking density. Construct B shows minimal force resistance, whereas Construct D presents intermediate performance with modest reinforcement. Overall, polyphenol incorporation modulates hydrogel network stiffness and extensibility, with tannic acid yielding the greatest mechanical enhancement.

The mechanical properties of Constructs B, C, and D were assessed via tensile testing to evaluate their potential for wound regeneration (Figure 5). Analyzing their stress–strain behavior allowed one to determine important characteristics including tensile strength, elongation at break, and Young′s modulus. The tensile test for Construct B revealed a peak force of 0.39 N and a break force of 0.05 N. The corresponding stress at peak was 0.07 MPa, with an elongation at break of 69.83. Young′s modulus, calculated from the initial linear region of the stress–strain curve, was found to be 0.0143 MPa. These results indicate that Construct B is highly flexible and can undergo significant deformation before breaking, making it suitable for applications requiring high elasticity and stretchability. Construct D exhibited a peak force of 0.39 N and a break force of 0.05 N. The stress at peak was 0.07 MPa, with an elongation at break of 23.29%. Young′s modulus, calculated from the initial linear region, was 0.0429 MPa, indicating a material with moderate stiffness and flexibility. The relatively low elongation at break compared with Construct B suggests that Construct D is less elastic but may provide better structural integrity under stress. This makes it suitable for applications where a balance between flexibility and mechanical strength is required. Construct C demonstrated a peak force of 3.19 N and a break force of 0.54 N. The stress at peak was 0.638 MPa, with an elongation at break of 45.34%. Young′s modulus, calculated from the initial linear region, was 0.2382 MPa, indicating a stiffer material compared with Constructs B and D. These results suggest that Construct C provides a balance between strength and flexibility, making it suitable for applications requiring moderate mechanical support.

### 3.2. Cytocompatibility and Proliferation Assays

**Figure 6:**
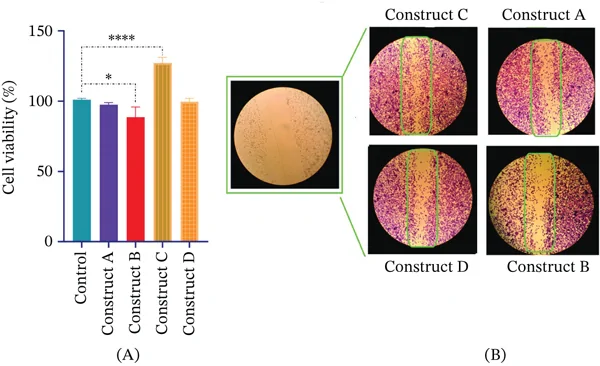
Cytocompatibility assessment of constructs. (A) Viability of human dermal fibroblasts after 24 h exposure to construct extracts (0.03 g/mL). (B) Scratch assay results (crystal violet staining), showing migration patterns. Staining results indicated that Construct C promoted closure of the scratch area compared with the control group. Data are presented as mean ± SD (*n* = 3). Significance:  ^∗^ *p* < 0.05;  ^∗∗∗^ *p* < 0.0001.

To assess the cytocompatibility of Constructs A, B, C, and D, an in vitro viability test (MTT assay) was performed on fibroblast cells (Figure 6A). The results of this assay showed that the highest cell viability was observed in cells treated with Construct C, which showed a cell viability rate of 127% following 24 h of incubation. This value significantly exceeded that of the control group and other tested groups (Construct D: 99.7%, Construct B: 88.7%, Construct A: 97.7%), highlighting the superior cytocompatibility of Construct C and its potential to support cellular activity. The increased viability percentage may indicate enhanced metabolic activity or cellular proliferation in response to Construct C, thereby emphasizing its potential for biomedical applications.

Also, a scratch assay was performed to further evaluate cell migration and proliferation, and crystal violet staining was applied to visualize cell migration into the scratched areas (Figure 6B). The staining results showed that Construct C significantly increased cell migration and proliferation in the wound area compared with the control group. These observations support the hypothesis that Construct C enhances fibroblast activity and has beneficial effects on cell growth and proliferation in response to treatment.

Findings from the MTT assay and scratch assay highlight the superior cytocompatibility of Construct C, as well as its ability to support enhanced fibroblast proliferation and migration.

### 3.3. Antibacterial Efficacy

**Figure 7:**
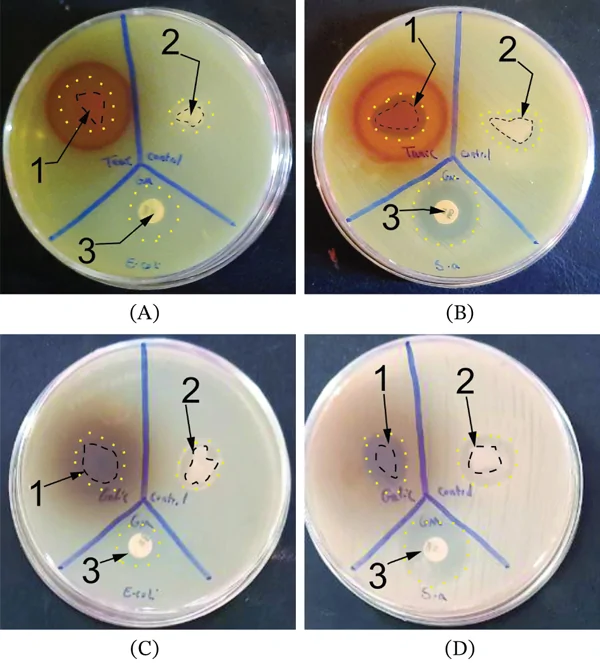
Antibacterial activity of constructs against *Escherichia coli* and *Staphylococcus aureus*. (A) Inhibition zones against *E. coli*: 1 (Construct C), 2 (Construct B), and 3 (gentamicin control). (B) Inhibition zones against *S. aureus*: same labeling as Figure 7A. (C,D) Analogous tests for Construct D. Yellow dotted lines: inhibition zones; black dashed lines: construct disks.

The antibacterial activity of the three main constructs (Constructs B, C, and D) was assessed against *S. aureus* ATCC 25923 (Gram‐positive) and *E. coli* ATCC 25922 (Gram‐negative) using the disk diffusion method (Figure 7). Zones of inhibition, indicating bacterial growth suppression, were measured in millimeters for each construct (B, C, and D), as well as for the gentamicin (20 *μ*g/disk).

Constructs B, C, and D exhibited varying degrees of antibacterial activity against both bacterial strains. The inhibition zones around each construct, as well as those for the controls (Construct B as baseline control and GM as positive control), are summarized in Table 2. The results indicate that all constructs effectively inhibited bacterial growth, with notable differences in their efficacy against Gram‐positive and Gram‐negative bacteria. Gentamicin served as a reference standard for antibacterial performance.

**Table 2:** Inhibition zones (millimeters).

Organisms	GM (20 *μ*g/disk)	Construct C/Construct B	Construct D/Construct B
*Staphylococcus aureus*	18	1.6	3
*Escherichia coli*	12	1.3	1.2

Abbreviation: GM, gentamicin.

### 3.4. In Vivo Wound Healing Studies

The effectiveness of the constructs in healing modeled wounds was assessed using the skin incision method. Wound closure rates and histological findings were analyzed to assess the healing process in the six groups. Prior to application to the incisional wounds, both sides of all hydrogel constructs were sterilized under a laminar flow hood by exposure to UV light for 30 min. All subsequent handling and application procedures were conducted under aseptic conditions to maintain sterility.

#### 3.4.1. Wound Closure Measurements

**Figure 8:**
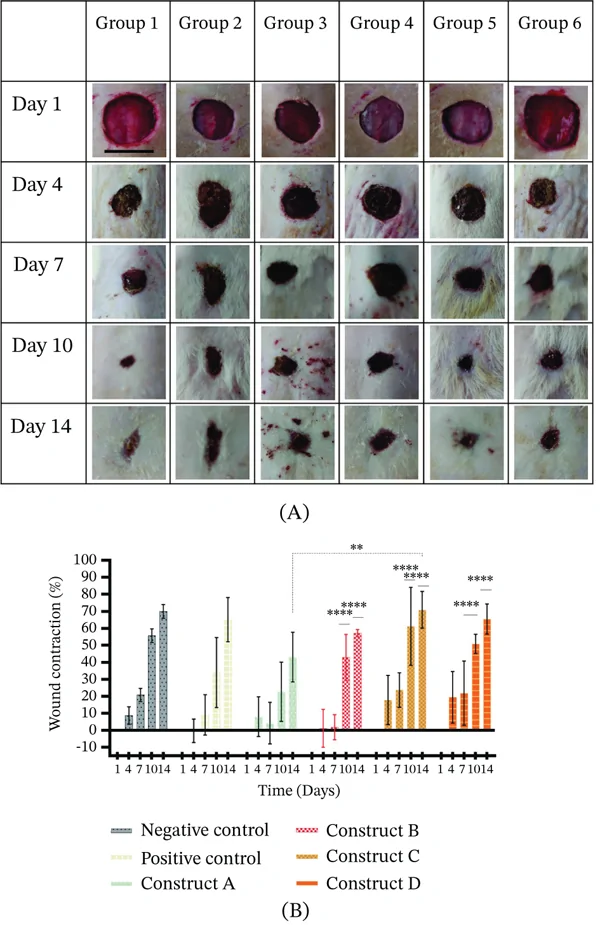
In vivo wound healing progression. (A) Macroscopic wound images at Days 1, 4, 7, 10, and 14. Groups: 1 (untreated), 2 (Hartmann Comfeel), 3–6 (Constructs A–D). (B) Quantitative wound closure rates. Data are presented as mean ±SD (*n* = 5–6). Statistical significance:  ^∗∗^ *p* < 0.01 and  ^∗∗∗∗^ *p* < 0.0001.  ^∗∗∗∗^ indicates a significant difference in wound closure rate between Days 14 and 1 within the respective group, whereas  ^∗∗^ indicates a significant difference in wound closure rate between Construct C and Construct A at Day 14.

The wound closure rate was calculated by analysis of images recorded on Days 1, 4, 7, 10, and 14. The captured images and their corresponding graph are shown in Figure 8A,B, respectively. In terms of changes in wound diameter, no significant difference was observed between the negative control (without wound dressing called Group 1) and the positive control (commercial wound dressing called Group 2), and wound closure occurred earlier in the negative control group (a statistically significant difference was observed in the negative control group on the 10th day compared with the first day, but for the positive control on the 14th day). Among the hydrogel‐treated groups, Constructs C (Group 5) and D (Group 6) exhibited superior wound closure, with over 65% of the wound area healed by Day 14. Construct A (Group 3) demonstrated a delayed healing process, with only 43% closure by Day 14, whereas Construct B (Group 4) achieved intermediate results, with 57% closure. The progressive reduction in wound size suggests that Constructs C and D effectively accelerated wound healing compared with other groups, even compared with the commercial Comfeel wound dressing.

#### 3.4.2. Histological Analysis

Histopathological examination of the skin of the wound area in the groups on Day 14 provided detailed insights into tissue regeneration. In the negative control group, epithelial regeneration was sparse, granulation tissue was minimal, and inflammatory cell infiltration remained extensive, indicating poor wound healing. The positive control group showed moderate epithelial regeneration, a mild reduction in inflammatory cells, and limited fibroblast activity. Constructs A and B (Groups 3 and 4) revealed partial epithelial regeneration, with moderate granulation tissue formation and fibroblast proliferation. However, Constructs C and D (Groups 5 and 6) demonstrated the most advanced histological outcomes, with well‐organized epithelial layers, substantial granulation tissue, and significantly higher fibroblast activity compared with other groups.

**Figure 9:**
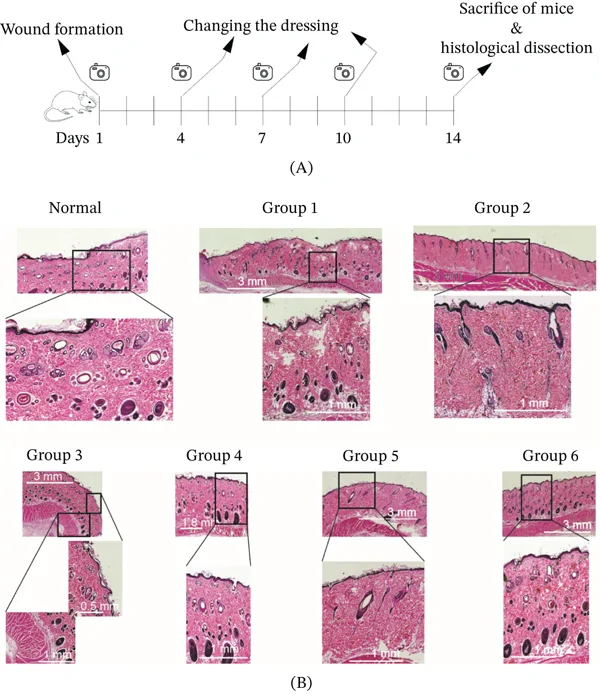
In vivo experimental timeline and histological assessment of wound healing. (A) Schematic of the in vivo wound healing study design. (B) Representative hematoxylin and eosin (H&E)–stained histological sections of wound tissue on Day 14. The negative control (Group 1) showed poor wound healing, with sparse epithelial regeneration, minimal granulation tissue, and extensive inflammatory infiltration. In contrast, Constructs A and B (Groups 3 and 4) exhibited partial tissue regeneration, whereas Constructs C and D (Groups 5 and 6) showed the most advanced healing, characterized by well‐organized epithelial layers, abundant granulation tissue, and increased fibroblast activity. Among all groups, Construct C exhibited the most advanced histological features of tissue regeneration among the tested groups.

Construct C exhibited the highest angiogenesis, as evidenced by an increased density of new capillaries within the granulation tissue, which was further corroborated by enhanced MT staining indicating robust collagen deposition. Construct D, although slightly less effective than Construct C in angiogenesis, showed pronounced fibroblast proliferation and a moderate reduction in inflammatory cell infiltration, suggesting a balanced healing profile. In contrast, Construct A showed limited angiogenesis and collagen organization, and Construct B displayed inconsistent histological features. A schematic diagram of the animal experiments is presented in Figure 9A, and the results of H&E staining are shown in Figure 9B.

##### 3.4.2.1. Inflammatory Cell Infiltration

Macrophages, neutrophils, and lymphocytes were assessed across groups. By Day 14, Constructs C and D showed the lowest neutrophil infiltration, indicative of lower inflammation and an enhanced resolution of the inflammatory phase. In contrast, the negative control group maintained high neutrophil and macrophage counts, representative of a chronic inflammation state. The positive control group and groups treated with Constructs A and B showed moderate reductions in inflammatory cell counts but lagged behind Constructs C and D in transitioning to the proliferative phase of healing.

##### 3.4.2.2. Collagen Deposition and Fibrosis

**Figure 10:**
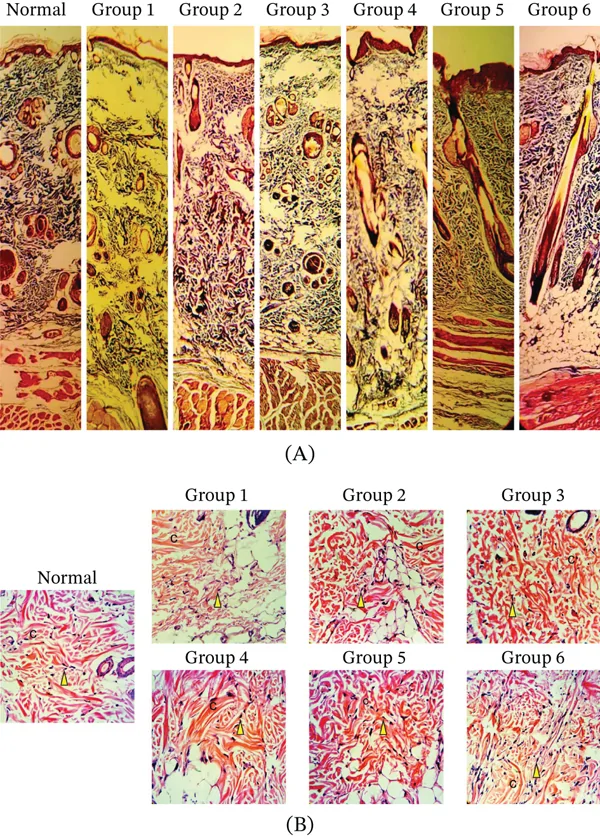
Collagen deposition in healed wounds (Masson′s trichrome staining). (A) Low‐magnification (40x) overview images. (B) High‐magnification (400x) views illustrating collagen fiber organization (blue staining). Masson′s trichrome staining revealed the most prominent and well‐organized collagen deposition in Constructs C and D (Groups 5 and 6), indicative of mature collagen deposition and tissue remodeling. Constructs A and B (Groups 3 and 4) displayed less organized collagen fibers and limited fibrosis compared with Constructs C and D, whereas the negative control group showed minimal collagen deposition, consistent with poor healing outcomes.

Collagen deposition was most prominent in Constructs C and D, as highlighted by MT staining, which revealed well‐organized collagen fibers. Constructs A and B displayed less organized collagen, with limited fibrosis. The negative control group exhibited minimal collagen deposition, consistent with inadequate healing. Images of MT staining are shown in Figure 10.

##### 3.4.2.3. Summary of Key Findings

The results indicate that Constructs C and D, incorporating LdECM, significantly enhance wound healing by promoting epithelial regeneration, reducing inflammation, and increasing angiogenesis and collagen deposition. Construct C, in particular, demonstrated the most balanced and effective wound healing profile, followed by Construct D. These findings underscore the potential of LdECM‐enriched hydrogels for advanced wound management applications.

## 4. Discussion

This study presents a bioactive wound‐dressing strategy based on the incorporation of decellularized ovine LdECM into a polymeric hydrogel network. As a biologically active component, LdECM is capable of providing structural proteins, GAGs, and matrix‐associated signaling molecules that play a role in tissue repair and offers biological functionality beyond that provided by conventional wound dressings, which primarily provide physical protection and moisture retention. The rationale for selecting LdECM is linked to the highly vascularized and regenerative nature of hepatic tissue, whose extracellular microenvironment is rich in basement membrane proteins such as laminin and fibronectin and is associated with signaling pathways involved in angiogenesis, cell–matrix interactions, and tissue regeneration [12, 13]. These characteristics are particularly relevant to wound healing, where rapid revascularization, fibroblast recruitment, and tissue remodeling are critical determinants of successful regeneration [12]. Furthermore, incorporation of TA or gallic acid not only improved the structural stability of the hydrogels through additional cross‐linking interactions but also contributed antioxidant and antimicrobial functionalities. Therefore, the combination of a bioactive LdECM hydrogel with a mechanically reinforced polymer matrix represents a biomimetic platform designed to integrate both biological signaling and structural support for enhanced wound repair. Unlike decellularized matrices obtained from primarily structural tissues, such as dermis, LdECM contains a complex microenvironment enriched with basement membrane proteins, including laminin and fibronectin, as well as GAGs capable of binding and presenting bioactive molecules to surrounding cells. Previous studies have demonstrated that LdECM hydrogels preserve key matrix components that support cell adhesion, migration, and tissue remodeling while maintaining a microenvironment favorable for endothelial and stromal cell function [16, 17, 23]. Furthermore, the liver possesses a remarkable intrinsic regenerative capacity and extensive vascular architecture, suggesting that its ECM may provide complementary proregenerative and proangiogenic signals derived from the microenvironment of a highly vascularized organ, thereby offering a distinct biological rationale for wound healing applications [12]. Although skin‐derived ECM represents a tissue‐matched alternative for cutaneous repair, LdECM may offer complementary biological signals associated with vascular remodeling and regeneration. Therefore, rather than serving solely as a structural scaffold, LdECM in the present study was incorporated as a bioactive component intended to enhance the regenerative potential of the polymeric wound dressing.

Before discussing the roles of TA, gallic acid, and the effects of LdECM, it is essential to examine the fundamental composition of the polymeric constructs. The FTIR results demonstrated effective integration of the polymers, with characteristic peaks corresponding to the functional groups of the constituent polymers (CMC, PEG, and SA). In the uncross‐linked hydrogels, a broad peak around 3390 cm^−1^, attributed to O–H stretching, indicated the presence of hydroxyl groups in CMC, PEG, and SA. This peak suggests the formation of hydrogen bonds between the polymer components, which is critical for the initial hydrogel formation [23, 24]. Peaks at approximately 2864 cm^−1^ (C–H stretching) correspond to alkyl groups in PEG and CMC, whereas the peak at 1597 cm^−1^ (C=O stretching) is associated with carboxylate groups in SA and CMC [25, 26]. These observations confirm the presence of all three polymers in the uncross‐linked hydrogel and highlight initial physical interactions through hydrogen bonding and electrostatic forces. After cross‐linking, significant changes were observed in the C=O and C–O–C regions. The blue shift of the asymmetric –COO^-^ stretching vibration from 1640 to 1650 cm^−1^ suggests ionic cross‐linking between the carboxylate groups of SA and Ca^2+^ ions [23]. This shift aligns with previous studies, which have shown that Ca^2+^ ions interact strongly with alginate carboxylate groups, leading to the formation of an “egg‐box” structure [26]. The red shift of the C–O–C ether bands of PEG (1105–1098 cm^−1^) implies that the PEG chains are effectively integrated into the polysaccharide matrix through intermolecular hydrogen bonding between the PEG ether oxygens and the –OH or –COOH groups of CMC and alginate. This interaction disrupts the crystalline packing of PEG, which is further supported by the broadening of the C–H stretching signals. The shift in the glycosidic linkage bands (1069–1061 cm^−1^) reflects a more rigid environment for the polymer backbone. As the cross‐link density increases, the pyranose rings of CMC and SA become more spatially restricted, altering the skeletal vibrational modes. This suggests a successful transition from a viscous polymer blend to a stable, three‐dimensional hydrogel network with improved mechanical integrity.

Importantly, these results align with those obtained from other tests, such as the swelling test, which will be discussed later. It is worth noting that the FTIR analysis was conducted to examine the cross‐linking of the base materials (CMC, SA, and PEG), whereas other parameters were evaluated in subsequent tests. For instance, the SEM test investigated the effects of TA and gallic acid on the microstructure of the wound dressing, with comparisons made in the presence and absence of LdECM gel.

Hydration control in cellulose‐based systems is reflected in their swelling behavior and is consistent with the role of network architecture in governing water uptake [27], as evidenced by our hydrogels. They exhibited an initial burst of water uptake (0–0.5 h), followed by rapid deswelling (0.5–2 h), and subsequently reached a stable plateau. Numerous polar sites, including hydroxyl and carboxyl groups belonging to CMC and LdECM, provided synergistic hydrophilicity, which contributed to the early swelling behavior [28, 29]. The higher swelling ratios of Constructs C and D may be attributed to the polyphenolic nature of TA and gallic acid, which promotes water uptake through hydrogen bonding and network interactions [30, 31]. The incorporation of TA and gallic acid may have altered polysaccharide–polyphenol interactions, contributing to a less compact network and enhanced diffusion [32]. Elevated equilibrium swelling resulting from hydrophilic phenolic domains has been previously reported, consistent with trends observed in TA‐based and gallic acid–based hydrogels [33, 34]. The subsequent relaxation of the polymer network and partial degradation of ionic cross‐links resulted in a deswelling phase. Hydrogen bonding and *π*–*π* interactions between polyphenols and polymer chains later stabilized the network [30, 32]. This balanced swelling–deswelling behavior, which enables rapid exudate absorption while preventing long‐term maceration, supports effective wound healing.

Rapid initial weight loss within the first 24 h was observed in the degradation profiles of Constructs B, C, and D, followed by a slower and more gradual degradation phase during the subsequent days. The base polymer network, Construct B, exhibited the greatest early mass loss, indicating limited structural stabilization of the polymer–ECM network in the absence of phenolic cross‐linking agents. Degradation behavior is a critical parameter in the design of hydrogel wound dressings, as it directly influences the duration of mechanical support and the persistence of the bioactive microenvironment at the wound site. In the present study, all constructs exhibited relatively rapid degradation, which is a common characteristic of hydrogels composed primarily of natural polymers and ECM components due to their high water content and susceptibility to hydrolytic and enzymatic breakdown [35, 36]. From a clinical perspective, excessively rapid degradation may reduce scaffold integrity and necessitate more frequent dressing replacement. However, complete long‐term persistence is not always desirable, as wound dressings are generally intended to provide temporary support while permitting progressive tissue remodeling and eventual scaffold resorption [37]. In the present work, wound dressings were replaced every 3 days during the in vivo study, which compensated for the relatively fast degradation of the hydrogel constructs while maintaining continuous wound coverage. Nevertheless, improving scaffold stability remains an important objective for future optimization. Incorporation of TA (Construct C) or gallic acid (Construct D) significantly moderated the degradation rate, demonstrating the stabilizing effect of phenolic cross‐linkers. Although degradation was not prevented, additional hydrogen bonding and intermolecular interactions likely increased resistance to hydrolytic and enzymatic degradation [38–40]. The slightly greater stability observed in gallic acid–containing hydrogels may be related to differences in molecular structure and cross‐linking density that influence water diffusion and degradation kinetics. These findings suggest that phenolic cross‐linking can partially overcome the inherent instability of ECM‐based hydrogels while preserving their biodegradability and biological functionality, both of which are desirable characteristics for wound healing applications.

The microstructural modulation of the hydrogels by both LdECM and polyphenols was evident from SEM analysis, with each component influencing the network in distinct ways. As extensively documented, the fibrillar nature of ECM increases porosity and enhances interconnectivity, which was clearly observed in our LdECM‐loaded constructs exhibiting highly open structures [41, 42]. In contrast, LdECM‐free hydrogels displayed more irregular and less interconnected pores. The TA‐induced pore size reduction reported in previous studies was also observed in our samples, indicating that TA acted as a strong polyphenolic cross‐linker, generating denser and more uniformly aligned networks [43]. Notably, in LdECM‐containing constructs, TA promoted both compaction and ECM‐driven porosity enhancement. On the other hand, GA produced smoother and moderately compact surfaces, reflecting weaker stabilizing interactions and identifying it as a milder cross‐linker with limited influence on pore morphology [44, 45]. Incorporation of GA into LdECM‐containing constructs resulted in hydrogels that were more porous while retaining smooth surface features.

Tensile analysis revealed pronounced effects of LdECM and polyphenolic agents on the mechanical behavior of the hydrogels. The control LdECM construct exhibited characteristics typical of natural hydrogels, including inherent softness and limited structural integrity, resulting in a compliant yet mechanically weak profile. Incorporation of TA produced a denser and stiffer network, accompanied by significant increases in tensile strength and elastic modulus. These enhancements indicate the formation of multiple hydrogen bonds and hydrophobic interactions between the phenolic groups of TA and the polymer chains. Previous studies have reported several‐fold improvements in mechanical strength following phenolic cross‐linking, often at the expense of reduced elongation. Consistent with these findings, the current research clearly demonstrated a conflict between flexibility and stiffness [33]. In contrast, hydrogels containing GA exhibited a more brittle behavior with limited extensibility. The modest mechanical reinforcement provided by GA is likely attributable to its fewer reactive sites and weaker cross‐linking capacity [34]. Based on these findings, TA may support load‐bearing and exudate‐resisting functions in wound‐dressing applications due to its superior mechanical reinforcement. However, excessive stiffness resulting from TA cross‐linking may limit adaptability to dynamic skin movements. Meanwhile, GA appears to contribute primarily to antioxidant functionality rather than mechanical enhancement.

The mechanical characterization of the constructs revealed a crucial interplay between structural integrity and bioactivity. Tensile testing demonstrated that LdECM incorporation generally improved the elasticity and resilience of the scaffolds, particularly in Construct C. However, SEM analysis showed that LdECM‐free Construct D initially exhibited a moderately compact surface matrix, which was disrupted upon LdECM addition. While this disruption improved porosity and biological accessibility, it compromised mechanical compactness. These findings highlight the inherent challenge of balancing mechanical strength and biological function in scaffold design [23, 46].

Cell viability of LdECM‐integrated hydrogels was significantly enhanced compared with LdECM‐free controls, as confirmed by MTT assays performed on human dermal fibroblasts. Construct A, which lacked both LdECM and polyphenolic agents, demonstrated baseline viability (~100%), highlighting the necessity of bioactive components to exceed inherent matrix effects. The LdECM alone was insufficient to fully mitigate degradation‐induced cellular stress, as evidenced by the near‐baseline viability observed in Construct B, thereby underscoring the additive benefit of polyphenol incorporation. Constructs C (LdECM + TA) and D (LdECM + GA) exhibited distinct effects of polyphenolic cross‐linking on cellular metabolism. Specifically, Construct C achieved the highest cell viability (~120%), whereas Construct D reached approximately 110%. Consistent with these findings, previous studies have reported high cytocompatibility for TA‐functionalized hydrogels, with TA demonstrating a supportive role in maintaining cell viability and minimal cytotoxicity [33]. Overall, these results indicate that polyphenol‐treated systems promote enhanced cellular responses. Similarly, fibroblast survival was improved in GA‐based hydrogels, corroborating the outcomes observed for Construct D [44].

Scratch assay results demonstrated that LdECM‐integrated hydrogels markedly enhanced fibroblast migration compared with LdECM‐free matrices. Construct A, which lacked both LdECM and bioactive components, exhibited minimal gap closure, underscoring the necessity of functional additives for effective cell motility. The capacity of LdECM to provide structural support and chemotactic cues was evident in Construct B (LdECM alone), which achieved an intermediate level of migration even in the absence of additional bioactive agents. In contrast, Construct C (LdECM + TA) showed rapid and nearly complete scratch closure. This pronounced migratory response is likely attributable to the synergistic effects of TA′s antioxidant activity and cross‐linking capability combined with ECM‐derived biochemical signals, collectively creating an optimized microenvironment for fibroblast motility. Construct D (LdECM + GA) produced a moderate enhancement in migration, exceeding that of the control but remaining below Construct C. This outcome may be related to the comparatively weaker cross‐linking capacity and antioxidant potential of gallic acid. Consistent with these observations, previous studies have shown that LdECM‐based hydrogels promote scratch closure by providing structural integrity and bioactive cues that facilitate fibroblast adhesion, directed migration, angiogenesis, and tissue regeneration [9, 47]. Likewise, TA accelerates scratch closure by stabilizing the scaffold, protecting against oxidative stress, and enhancing fibroblast adhesion and motility during early wound healing stages [23, 46]. Moreover, GA, as a phenolic compound, contributes to wound repair by mitigating oxidative stress, enhancing fibroblast and keratinocyte migration, and accelerating re‐epithelialization [48, 49]. It is important to note that the scratch assay offers a preliminary assessment of fibroblast migration and proliferation. However, this method does not discriminate between migratory and proliferative contributions to wound closure. Therefore, more advanced approaches, such as live‐cell imaging, proliferation‐specific markers, or molecular signaling analyses, are required to elucidate the precise underlying mechanisms.

Antibacterial evaluation demonstrated that all LdECM‐containing hydrogel constructs inhibited the growth of *S. aureus* and *E. coli*, although the magnitude of inhibition varied among formulations. Construct B exhibited only weak antibacterial activity, likely reflecting the limited intrinsic antimicrobial properties of the CMC/SA/LdECM matrix and its primary function as a physical barrier to microbial invasion [50]. In contrast, incorporation of TA (Construct C) significantly enhanced antibacterial performance, particularly against *S. aureus*, consistent with previous reports describing TA‐mediated bacterial growth inhibition through protein complexation and membrane interactions [33]. Construct D exhibited the strongest antibacterial effect, in agreement with studies demonstrating the broad‐spectrum antimicrobial activity of gallic acid–containing biomaterials [51]. These findings suggest that LdECM primarily serves as a bioactive structural matrix, whereas phenolic additives provide complementary antimicrobial functionality, thereby enhancing the overall therapeutic potential of the wound dressing.

The biological outcomes reinforce the important role of LdECM in promoting tissue regeneration during wound healing. Constructs C and D exhibited superior wound closure, epithelial regeneration, granulation tissue formation, and vascularization compared with LdECM‐free constructs. Interestingly, although the negative control group demonstrated wound closure rates comparable with those observed in Constructs C and D, histological examination revealed substantial differences in tissue quality. It is well established that wound healing in rodents occurs predominantly through wound contraction, which can rapidly reduce wound area without necessarily restoring normal tissue architecture and function [52, 53]. Therefore, wound closure alone should not be considered a sufficient indicator of successful tissue regeneration. In the present study, untreated wounds displayed less organized tissue architecture, whereas LdECM‐containing constructs promoted the formation of well‐developed epidermal and dermal layers, improved collagen organization, and enhanced neovascularization. These findings suggest that the beneficial effects of LdECM‐based hydrogels extend beyond simple wound contraction and involve active modulation of the regenerative microenvironment. Previous studies have shown that LdECM retains structural proteins, GAGs, and matrix‐associated bioactive molecules that support cell adhesion, migration, ECM remodeling, and angiogenesis [16, 17]. Furthermore, Bobrova et al. demonstrated that the addition of decellularized liver microparticles to silk fibroin scaffolds improved fibroblast adhesion and proliferation and significantly accelerated wound healing by completely regenerating skin layers and hair follicles [15]. Such mechanisms may explain the superior histological outcomes observed in the LdECM‐containing groups of the present study. Notably, Construct C exhibited the greatest angiogenic response, suggesting that the combination of LdECM‐derived bioactivity and TA‐mediated scaffold stabilization created a microenvironment favorable for vascular maturation and tissue remodeling. Collectively, these findings indicate that evaluation of wound healing should incorporate both wound closure and tissue quality parameters, as the regeneration of structurally organized and vascularized tissue represents a more meaningful indicator of successful repair than wound contraction alone [52, 53].

Notably, the combination of LdECM and TA in Construct C resulted in the highest angiogenesis and wound healing efficacy, further underscoring the synergistic benefits of these bioactive components. Future studies could explore advanced fabrication techniques, such as coelectrospinning, to improve ECM integration and scaffold homogeneity [36, 37].

Histological analysis provided compelling evidence of the constructs′ regenerative potential. Constructs C and D demonstrated well‐organized epithelial layers, increased fibroblast activity, and enhanced angiogenesis, validating their bioactive design. However, the slightly lower angiogenesis observed in Construct D compared with Construct C suggests that ECM′s structural heterogeneity alone may not fully offset the limitations posed by reduced porosity. The supplementation of ECM with angiogenic factors like VEGF could further enhance capillary formation and improve scaffold efficacy [54].

The immunomodulatory effects of the hydrogels were also noteworthy. Constructs C and D significantly reduced neutrophil and macrophage infiltration by Day 14, indicating a transition from an inflammatory to a proliferative healing phase. This aligns with findings that ECM promotes macrophage polarization toward a prohealing phenotype [55, 56]. However, the long‐term immune response to these constructs remains unexplored. Future investigations should consider incorporating immunomodulatory agents, such as interleukin‐10, to further optimize the anti‐inflammatory properties of these scaffolds [57].

Despite these promising results, standardization of ECM preparation and scaffold synthesis remains a critical challenge. Variability in decellularization protocols can lead to batch‐to‐batch inconsistencies, potentially affecting scaffold quality and clinical reliability. Recent studies have highlighted the potential of automated decellularization systems and computational monitoring tools to improve process reproducibility, optimize decellularization efficiency, and facilitate objective assessment of ECM preservation [10, 58, 59]. Therefore, integration of automated manufacturing and quality control strategies may represent an important step toward the large‐scale production and clinical translation of ECM‐based wound dressings [60].

This study presents a pioneering strategy for wound care by integrating ovine LdECM into bioactive hydrogels. By leveraging the inherent bioactivity of ECM alongside structural support from hydrophilic polymers such as CMC and SA, these constructs offer a promising platform for advanced wound dressings. The incorporation of additional cross‐linking agents like TA and gallic acid further enhances antimicrobial activity, inflammation resolution, and tissue regeneration. Constructs demonstrated superior mechanical stability, optimized degradation profiles, and enhanced biological activity, as confirmed by in vitro and in vivo analyses. Constructs C and D, particularly, exhibited significant improvements in fibroblast proliferation, angiogenesis, and epithelial regeneration, validating their potential for wound healing applications.

Despite the encouraging findings, several limitations of this study should be acknowledged. First, the in vivo experiments were conducted using a relatively limited number of animals, and larger studies are needed to further validate the reproducibility and robustness of the observed therapeutic effects. Second, although incorporation of TA and gallic acid improved hydrogel stability, the constructs still exhibited relatively rapid degradation, requiring dressing replacement every 2 days during the wound healing study. In addition, whereas the regenerative effects of LdECM were supported by enhanced wound closure and histological outcomes, the retention of specific growth factors and the molecular mechanisms responsible for its proregenerative activity were not directly investigated. The antibacterial assessment was limited to an agar diffusion assay and therefore may not fully reflect antimicrobial performance under physiological conditions. Furthermore, translation of LdECM‐based biomaterials to large‐scale applications will require further optimization and standardization of decellularization and manufacturing processes to minimize batch‐to‐batch variability. Future studies should focus on mechanistic characterization of LdECM bioactivity, long‐term safety evaluation, and validation in larger preclinical models.

Overall, the present findings demonstrate that incorporation of LdECM into a polymeric hydrogel network can provide a bioactive wound‐dressing platform with favorable physicochemical properties, cytocompatibility, antibacterial activity, and enhanced wound healing performance in vivo. The addition of TA and gallic acid improved scaffold stability and contributed supplementary antimicrobial functionality, while maintaining the biological advantages associated with LdECM. Although further optimization and mechanistic studies are required, the results support the potential of LdECM as a biologically active component for the development of next‐generation wound dressings.

## Author Contributions

Hadis Doosti and Parisa Mohammadi contributed equally to this work.

## Funding

This work was supported by Kermanshah University of Medical Sciences (10.13039/501100005317, IR.KUMS.AEC.1403.012).

## Conflicts of Interest

The authors declare no conflicts of interest.

## Data Availability

The data that support the findings of this study are available from the corresponding author upon reasonable request.
